# Preparation of Tea Tree Oil/Poly(styrene-butyl methacrylate) Microspheres with Sustained Release and Anti-Bacterial Properties

**DOI:** 10.3390/ma11050710

**Published:** 2018-05-01

**Authors:** Guanquan Lin, Huayao Chen, Hongjun Zhou, Xinhua Zhou, Hua Xu

**Affiliations:** 1 School of Chemistry and Chemical Engineering, Zhongkai University of Agriculture and Engineering, Guangzhou 510225, China; LG752492434@126.com (G.L.); huayao_011@163.com (H.C.); dlutxuhua@163.com (H.X.); 2 Guangzhou Key Lab for Efficient Use of Agricultural Chemicals, Guangzhou 510225, China

**Keywords:** tea tree oil, microspheres, sustained release, anti-bacterial effect

## Abstract

Using butyl methacrylate (BMA) and styrene (St) as monomers and divinylbenzene (DVB) as a crosslinking agent, P(St-BMA) microspheres were prepared by suspension polymerization. Tea tree oil (TTO) microspheres were prepared by adsorbing TTO on P(St-BMA) microspheres. The structure and surface morphology of P(St-BMA) microspheres and TTO microspheres were characterized by Fourier transformed infrared spectroscopy (FTIR), optical microscopy, and Thermogravimetric analysis (TGA). In doing so, the structural effect of P(St-BMA) microspheres on oil absorption and sustained release properties could be investigated. The results show that the surface of the P(St-BMA) microspheres in the process of TTO microsphere formation changed from initially concave to convex. The TTO microspheres significantly improved the stability of TTO, which was found to completely decompose as the temperature of the TTO increased from about 110 °C to 150 °C. The oil absorption behavior, which was up to 3.85 g/g, could be controlled by adjusting the monomer ratio and the amount of crosslinking agent. Based on Fickian diffusion, the sustained release behavior of TTO microspheres was consistent with the Korsmeyer-Pappas kinetic model. After 13 h of natural release, the anti-bacterial effect of the TTO microspheres was found to be significantly improved compared to TTO.

## 1. Introduction

In recent years, consumers have paid increasing attention to food safety, as the quality of ingested food is generally directly related to human health. In the process of food production, food may get contaminated by various microbial species [1]. The statistics from the Center for Disease Control (CDC) estimate that about 420,000 people die from various foodborne diseases every year [2]. *Escherichia coli* (*E. coli*), particularly *E. coli* O157:H7, refers to a pathogenic bacterium and is one of the main microorganisms that contaminate food, resulting in the production of toxic compounds [3,4]. The bacterial strain has been found in the production of many foods, including milk and meat. If food contaminated with *E. coli* enters the body, conditions such as diarrhea, cholera, typhoid, and other intestinal diseases result, which can potentially lead to severe sepsis. Furthermore, beef contaminated with *E. coli* bacteria may also contain the Shiga toxin (Stx). Symptoms of infection after eating often include nausea, vomiting, abdominal cramps, bloody stools, and even renal failure [5]. Therefore, it is critically important to prevent microbial contamination in food production. The use of anti-bacterial agents represents one of the most effective ways to inhibit bacterial growth and prevent microbial contamination. In practice, chemical anti-bacterial agents are usually added to food directly. Although the bactericidal effect is rapid, the anti-bacterial agent may cause certain carcinogenicity, teratogenicity, and chronic toxicity problems. However, the highly active anti-bacterial agent is attached to the polymer material and packaged into bags [6]. Using fumigation, the agent kills not only bacteria on the food surface, but also microbes in space. To ensure food safety and hygiene, the anti-bacterial agents and polymers do not come into direct contact with food [7,8]. Therefore, it is important to select a safe anti-bacterial agent and polymer material with good sustained-release properties for the preparation of this anti-bacterial material.

Tea tree oil (TTO) [9,10], a volatile hydrophobic liquid, is produced by the secondary metabolism of plants. It has been shown to be non-toxic, non-irritating, and non-corrosive to humans [11,12]. On the other hand, TTO exhibits a wide spectrum of anti-bacterial, anti-viral, and anti-inflammatory activities. Its wide range of antimicrobial activity is mainly due to its diversity of components, especially its volatile constituents. The volatile compounds in TTO are mainly 1,8-cineole, terpinen-4-ol, and α-terpilenol, all of which exhibit antimicrobial activity [13]. 1,8-cineole has been shown to destroy the cell menbrane of *E. coli,* while terpinen-4-ol exhibits strong antibacterial, disinfection, and anti-corrosive effects. α-terpilenol has been demonstrated to feature suitable permeability and exhibits a killing effect on common pathogens such as *Staphylococcus aureus*, *E. coli*, pseudomonas aeruginosa, candida aibicans, etc. [14,15]. TTO is not harmful to the human body and has a suitable, natural anti-bacterial effect; thus, it may be used as an anti-bacterial agent in food. However, its oxygen-, light-, or temperature-sensitive characteristics greatly affect its overall applicability [16,17]. Cui et al. [18,19] have used beta-cyclodextrin (β-CD) to capture TTO and form TTO/β-CD aggregates exhibiting high encapsulation efficiency and significantly improved chemical stability of TTO. In addition, TTO/β-CD has been encapsulated into a poly(ethylene oxide) (PEO) matrix by electrostatic spinning. TTO as the active anti-bacterial agent can inhibit the growth of *E. coli* to prolong the shelf-life of beef. Simultaneously, after coating, the sustained release time of TTO was prolonged significantly at different temperatures (4 °C, 12 °C, 25 °C, and 35 °C). This may have greatly improved the anti-bacterial effect and application value of TTO. Chen et al. [20] have used sodium alginate (SA) and a quaternary ammonium salt of chitosan (HACC) for the synthesis of TTO-loaded anti-bacterial microcapsules by complex coacervation. These microcapsules were demonstrated to improve the thermal stability and prolong the sustained release time of TTO, ultimately resulting in a sustained anti-bacterial effect against *Staphylococcus aureus* (*S. aureus*) and *E. coli*. Although the above examples may solve stability and release problems of TTO, the preparation process is rather complex, often involving heating processes that may accelerate the volatilization of the effective components in TTO. However, the preparation of oil absorption microspheres to help adsorb TTO may solve these shortcomings [21].

Polyacrylate crosslinked microspheres represent a new type of oil-absorbing material, as they exhibit powerful oil absorption properties with negligible oil leakage. Furthermore, the structure of the microspheres can be easily adjusted, which may further help regulate the release of volatile oils [22]. At present, the research on the combination of oil-absorbing microspheres with essential oil and their application to food-inhibiting bacteria is relatively rare. In this paper, rigid monomer St and flexible monomer BMA were selected, and P(St-BMA) microspheres were prepared via suspension polymerization. The microspheres adsorbed TTO to form TTO microspheres. The structure, morphology, and particle size were measured, and the thermal stability, absorption performance, sustained release performance, and sustained anti-bacterial performance were studied in detail. The obtained results showed that the TTO microspheres offer great potential in the field of anti-bacterial agents.

## 2. Results and Discussion

### 2.1. Structure and Morphology Analysis

**Figure 1:**
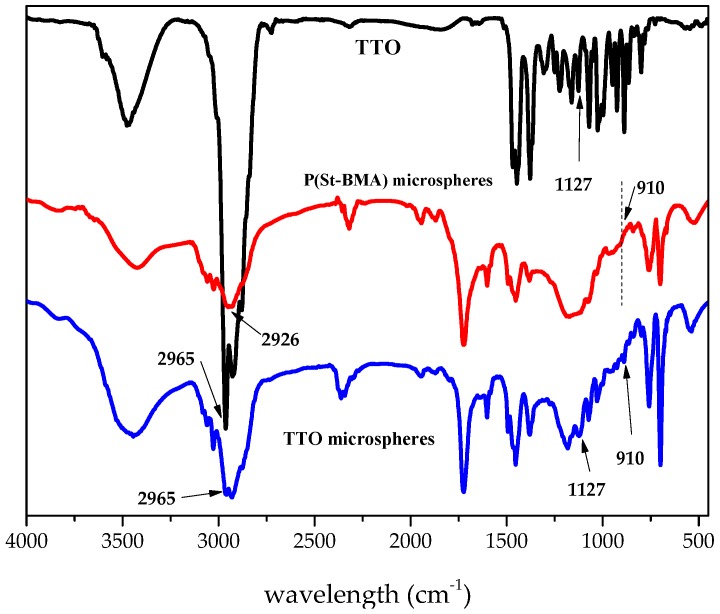
FTIR spectra of TTO, P(St-BMA) microspheres, and TTO microspheres.

Figure 1 and Table 1 show the FTIR spectra and absorption peaks of TTO, P(St-BMA) microspheres, and TTO microspheres. For P(St-BMA) microspheres, the strong peak observed at 688 cm^−1^ and 763 cm^−1^ corresponded to the characteristic absorption signals of monosubstituted benzene. The peaks at 2926 cm^−1^, 1450 cm^−1^, and 1600 cm^−1^ could be ascribed to the stretching vibration of the C–H and C–C bonds of the benzene ring, respectively. The peak at 1729 cm^−1^ corresponded to the carbonyl stretching vibration absorption peak. There was no obvious absorption peak near 910 cm^−1^, indicating that the C=C bond of St and BMA were already cleaved. For TTO, the stretching vibration peak corresponding to the C–H bond appeared at 2965 cm^−1^. The peak at 1127 cm^−1^ was ascribed to the stretching vibration absorption peak of the C–O bond of the tertiary alcohol in terpenes and terpineol, respectively. The peak at 910 cm^−1^ corresponded to the bending vibration absorption peak of an unsaturated double bond. For TTO microspheres, the peak at 1729 cm^−1^ corresponded to the characteristic peak of TTO [23], covered by a carbonyl absorption peak of TTO microspheres. Compared to P(St-BMA) microspheres, three new absorption peaks for TTO microspheres appeared at 2965 cm^−1^, 1127 cm^−1^, and 910 cm^−1^, corresponding to the characteristic peaks of TTO and further indicating that TTO was adsorbed by the P(St-BMA) microspheres.

**Figure 2:**
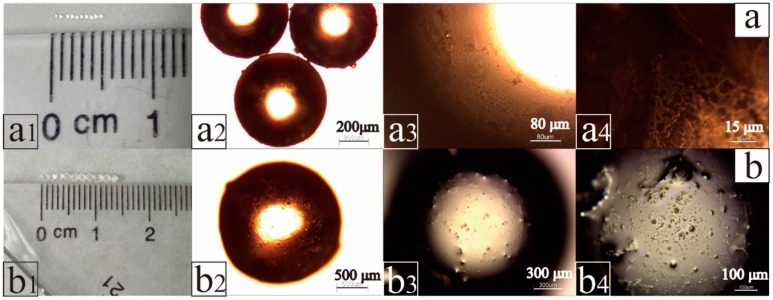
Surface topography of P(St-BMA) microspheres (**a**) and TTO microspheres (**b**).

Figure 2(a_1_,b_1_) shows particle size diagrams of microspheres before and after TTO absorption. Figure 2a_1_ shows that 10 P(St-BMA) microspheres were 0.5 mm long, and the average particle size was about 500 μm. As shown in Figure 2b_1_, after adsorption of TTO the length of 10 TTO microspheres was approximately 14.5 mm, and the average particle size was about 1450 μm. Figure 2(a_2_,b_2_) shows the morphology of P(St-BMA) microspheres and TTO microspheres in an optical microscope. The P(St-BMA) microspheres and TTO microspheres exhibited a spherical shape, and the particle size of the two corresponded to the data shown in Figure 2(a_1_,b_1_). As shown in Figure 2(a_3_,a_4_), the surface of P(St-BMA) microspheres was rough and concave, because the monomers of St and BMA cross-linked to form a three-dimensional network scaffold in the presence of a DVB crosslinking agent. As shown in Figure 2(b_3_,b_4_), compared to the P(St-BMA) microspheres, the surface of TTO microspheres was convex instead of concave. Most likely, this latter finding was due to the three-dimensional network of the P(St-BMA) microspheres forming a porous structure. Furthermore, the density difference between air and TTO was large and the surface tension of TTO was low. As a result, during adsorption of TTO by the P(St-BMA) microspheres, part of the air inside the pores was not completely consumed, resulting in the formation of a liquid film on the surface.

### 2.2. Thermal Stability Analysis

**Figure 3:**
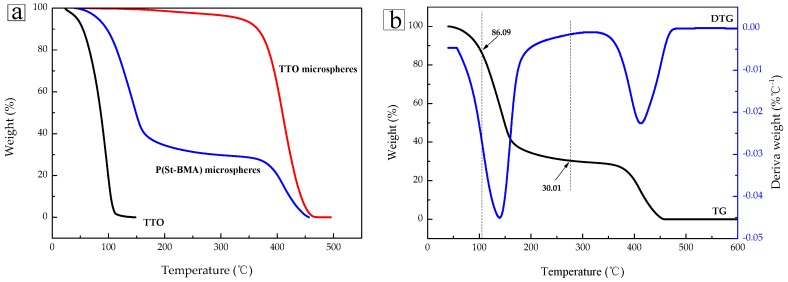
(**a**) TGA curves of TTO, P(St-BMA) microspheres, and TTO microspheres, and (**b**) TGA and DTG curves of the TTO microspheres.

Figure 3a shows the results from TG analysis of TTO, P(St-BMA) microspheres, and TTO microspheres. As shown in the TGA curve of TTO, the mass fraction of TTO was 0 at 110 °C, and the TTO proved to be highly volatile and further decomposed at a temperature range of 40 °C to 110 °C. The latter finding indicates that TTO featured poor thermal stability. As shown in the curve of the P(St-BMA) microspheres, two weight loss peaks could be observed. The first peak corresponds to the weight loss peak of complete evaporation and decomposition of TTO. The second peak corresponds to the weight loss peak after decomposition of the P(St-BMA) microspheres. To further investigate the condition of weight loss, the DTG of TTO microspheres was studied. As shown in Figure 3b, the TTO microspheres volatized and decomposed completely at 270 °C. At this temperature, the remaining mass fraction of TTO was 30.01%, and it accounted for 69.99% of the total quantity. At 110 °C, the total mass fraction of TTO was 86.09%, and the weight loss rate was 13.91%. Thus, the overall weight loss rate of TTO was only 19.87%. Upon comparison, it becomes clear that the stability of TTO after adsorption to the P(St-BMA) microspheres was significantly improved. The latter finding is most likely due to the fact that the P(St-BMA) microspheres, which began to decompose at 360 °C, exhibited suitable thermal stability. The material was therefore able to provide good physical protection for TTO and further prevented TTO from volatizing and decomposing by direct heating. On the other hand, the underlying force of the P(St-BMA) microspheres to adsorb TTO mainly involved Van der Walls forces and solvation between the lipophilic group of the P(St-BMA) microspheres and the TTO [24]. Taken in concert, the results described above outline why the TTO microspheres were able to significantly improve the stability of TTO.

### 2.3. Oil Absorption Performance Analysis

**Figure 4:**
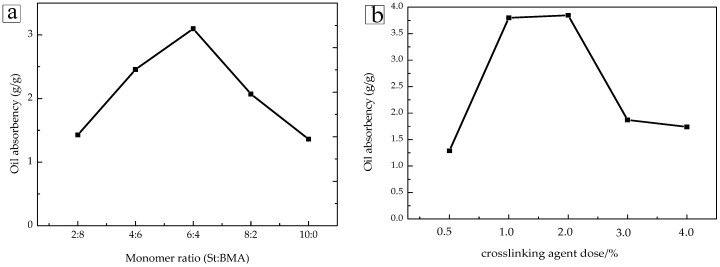
(**a**) Oil absorption characteristics of the P(St-BMA) microspheres at different monomer ratios and (**b**) oil absorption characteristics of the P(St-BMA) microspheres at different dosages of the DVB crosslinking agent.

Figure 4a shows the oil absorption rate of the P(St-BMA) microspheres at different monomer ratios. When the monomer ratio was 2:8, the oil absorption rate was 1.45. In addition, when the monomer ratio was 6:4, the oil absorption rate reached a maximum. As the amount of BMA with flexible long chain lipophilic groups became too large, the structure was easily dissolved in the hydrophobic phase, resulting in disruption of the three-dimensional network structure. More importantly, in the presence of too many long-chain groups, the three dimensional network structure was found to be relatively “loose”, reducing the dissolution properties in hydrophobic media [25]. This latter finding is the reason why the oil absorption ratio was low when the BMA ratio was high. However, St exhibiting a rigid benzene ring structure could improve the rigidity of the three-dimensional network structure, thereby enhancing the oil absorption performance. Therefore, by adjusting the flexible structure and the rigid structure for BMA and St, the oil absorption rate reached a maximum at a monomer ratio of 6:4. However, the oil absorption rate decreased as the St monomer ratio continued to increase. This latter finding was due to the microspheres exhibiting too many rigid groups, leading to a structure that was too rigid, resulting in a three-dimensional network structure that was too tight, ultimately reducing the oil absorption rate.

Figure 4b shows the oil absorption rate of P(St-BMA) microspheres at various amounts of DVB crosslinking agent. The oil absorption rate first increased and then decreased as the amount of DVB increased. When the amount of DVB was in the range of 0.5% to 2.0%, the three-dimensional network structure of the microspheres was found to be loose to moderate, resulting in a great increase in the oil absorption rate. According to the Flory theory [26], after reaching a certain degree, the oil absorption rate was inversely proportional to the crosslinking density. As the amount of DVB increased further, the oil absorption rate decreased. This result was mostly due to the crosslinking density that further increased due to the three-dimensional network structure that was too tight, which was not favorable to the adsorption of TTO.

### 2.4. Sustained-Release Performance Analysis

**Figure 5:**
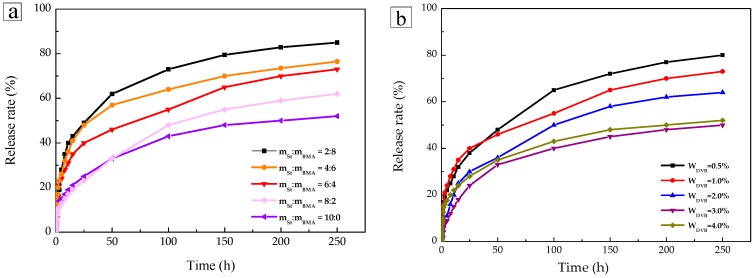
(**a**) Sustained release properties of the TTO microspheres at different monomer ratios and (**b**) sustained release properties of TTO microspheres at various dosages of the DVB crosslinking agent.

Figure 5a shows the sustained release properties of TTO microspheres at various monomer ratios. The sustained release curve shows a quick release trend at first, with a slower release thereafter. At the beginning of the first 25 h, the TTO release rate was fast, most likely caused by the volatilization of the TTO on the surface of the microspheres. As shown, a faster release rate could be observed using a larger proportion of BMA. The reason for this finding was due to BMA featuring flexible long chain lipophilic groups that may increase the storage space for hydrophobic molecules on the P(St-BMA) microspheres. Furthermore, the porosity of the three-dimensional network structure increased, and therefore the intermolecular forces and diffusion resistance were found to be reduced, ultimately leading to an increased TTO release rate. In addition, the larger the St ratio, the better the sustained release performance. Because St, exhibiting a rigid benzene ring structure, could increase the tightness of the three-dimensional network structure of the microspheres, the TTO release rate could be slowed down, improving the overall sustained release performance. However, this feature was not favorable to TTO absorption due to the increased tightness of the three-dimensional network structure.

Figure 5b shows the sustained release properties of TTO microspheres at various amounts of the DVB crosslinking agent. The sustained release performance of the TTO microspheres improved as the amount of the DVB crosslinking agent increased. The amount of DVB determined the degree of cross-linking to determine the tightness of the three-dimensional network structure. With increasing tightness, intermolecular forces, and diffusion resistance, an impeding effect on the TTO release rate was observed, leading to the slowing down of the TTO release and the general improvement of the sustained-release properties.

In order to further study the sustained release mechanism of the TTO microspheres, the data of different monomer ratios were fitted to the kinetic models of Zero-order [27], First-order [28], Higuchi [29], and Korsmeyer-Pappas [30], respectively. The corresponding results are shown in Table 2. The most consistent model for this sustained release behavior was the Korsmeyer-Pappas kinetic model. When the ratio of m_St_:m_BMA_ was 2:8, 4:6, 6:4, 8:2, and 10:0, the release indices *n* were 0.3418, 0.4446, 0.4191, 0.3275, and 0.4005, respectively. The indices *n* were less than 0.45, indicating that the TTO release was mainly based on Fickian diffusion [31,32].

### 2.5. Antimicrobial Activity Analysis

**Figure 6:**
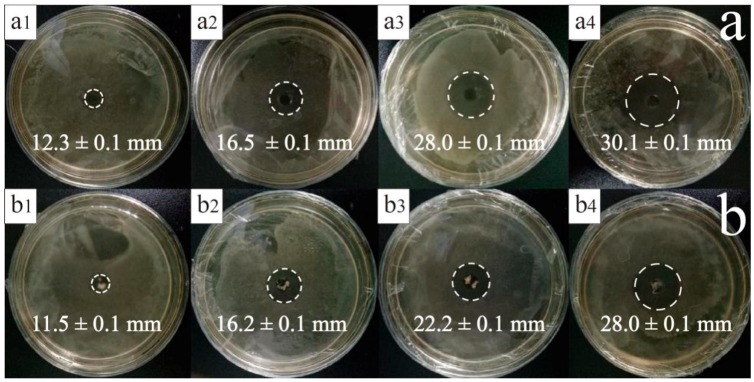

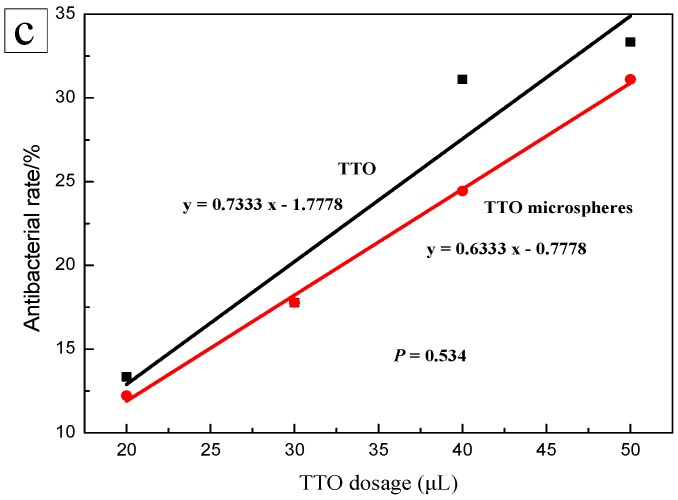
(**a**) Inhibition zones of TTO at various dosages 20 μL (**a_1_**), 30 μL (**a_2_**), 40 μL (**a_3_**), and 50 μL (**a_4_**) for 24 h; (**b**) inhibition zones of TTO microspheres at various dosages 20 μL (**b_1_**), 30 μL (**b_2_**), 40 μL (**b_3_**), and 50 μL (**b_4_**) for 24 h; and (**c**) anti-bacterial rate of TTO and TTO microspheres at various dosages.

As shown in Figure 6a,b, the diameter of the inhibition zone increased as the amount of TTO increased. Furthermore, the anti-bacterial rate was proportional to the TTO amount. The inhibition zone of the TTO microspheres was smaller than that of TTO due to the fact that TTO microspheres generally serve as an inhibitor and have further been reported to slow down the release of terpineol [33], 1,8-cineole, and other substances [34]. Although the inhibition zone of the TTO microspheres is small, the diameter does not differ to a significant extent. As shown in Figure 6c, the slope *K* of TTO was 7.3333, and the *K* of TTO microspheres was 6.3333. As the slope of the two was relatively close and the probability value *P* = 0.534 was higher than the critical statistical value (*P* = 0.05) by covariance analysis, with a low TTO amount, the anti-bacterial effect of the two was not significantly different. With a larger TTO amount, the TTO anti-bacterial effect improved. However, as shown in Figure 6b_4_, the anti-bacterial effect of the TTO microspheres was still significant, even at low TTO amounts.

**Figure 7:**
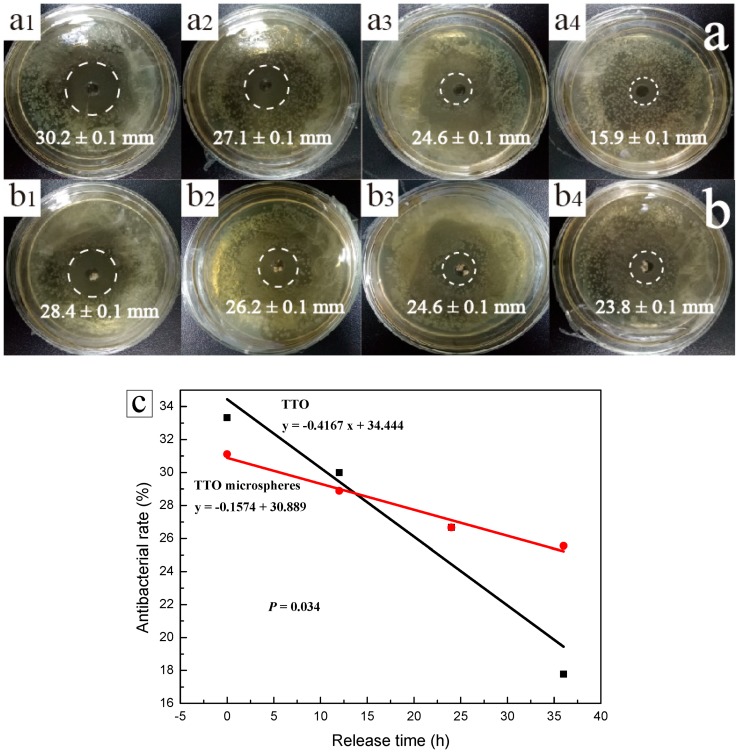
(**a**) Inhibition zones of TTO at various release times 0 h (**a_1_**), 12 h (**a_2_**), 24 h (**a_3_**), and 36 h (**a_4_**); (**b**) inhibition zones of TTO microspheres at various release times 0 h (**b_1_**), 12 h (**b_2_**), 24 h (**b_3_**), and 36 h (**b_4_**); and (**c**) anti-bacterial rate of TTO and TTO microspheres at various release times.

In order to further study the sustained release properties of TTO and TTO microspheres, both materials were released at different time points as part of this anti-bacterial experiment. The corresponding results are shown in Figure 7a,b. After a release time of 36 h, the inhibition zone of TTO changed from about 30.2 mm to 15.9 mm. However, the inhibition zone of the TTO microspheres was only reduced from 28.4 mm to 23.8 mm. Figure 7(b_2_–b_4_) shows little change in the inhibition zone, indicating that the anti-bacterial effect of TTO microspheres was better than that of TTO. In order to further understand the relationship between the anti-bacterial rate and the release time, the corresponding fitting curve is shown in Figure 7c. The slope *K* of the TTO was −0.4167, while that of the TTO microspheres was −0.1574. This latter finding shows that the anti-bacterial effect of the TTO rapidly decreased upon increasing the release time. As the *P* = 0.034 was lower than the critical statistical value (*P* = 0.05), the anti-bacterial effect of the two was significantly different. Both intersect at 13 h, indicating that the anti-bacterial effects of the two materials are the same at this time. However, after 13 h, the anti-bacterial effect of the TTO microspheres was better than that of TTO. This finding demonstrated that TTO microspheres were able to continuously release TTO and continuously inhibit the growth of *E. coli*.

## 3. Materials and Methods

### 3.1. Materials

BMA, St, and 2,2′-azobisisobutyronitrile (AIBN) were obtained from Tianjin Damao Chemical Reagents (Tianjin, China). Polyvinyl alcohol and DVB were purchased from Shanghai Aladdin Biochemical Technology Co. Ltd. (Shanghai, China). TTO was obtained from Jiangxi Taicheng Natural Flavor Co., Ltd. (Jian, China). All chemicals obtained from commercial sources were analytically pure and used as received without further purification.

### 3.2. Preparation of TTO Microspheres

**Figure 8:**
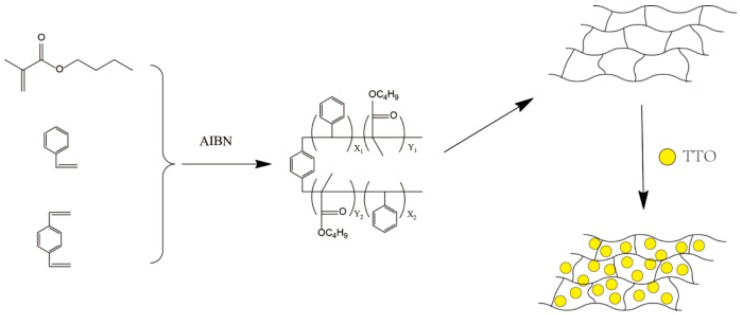
Schematic representation for the synthesis of TTO microspheres.

1.2 g of polyvinyl alcohol and 60 mL of deionized were placed in a 250 mL three-necked flask with mechanical stirring at 300 r/min and heated at 80 °C for 1 h. 12 g of St, 8 g of BMA, 0.6 g of DVB, and 0.2 g AIBN were mixed and added to the flask with stirring at 500 r/min for 6 h. P(St-BMA) microspheres were obtained upon filtration, washed, and dried. Finally, a certain amount of TTO was adsorbed by P(St-BMA) microspheres to obtain TTO microspheres. The overall preparation process is outlined in Figure 8.

### 3.3. Structural Characterization of TTO Microspheres

The structures of P(St-BMA) microspheres and TTO microspheres were analyzed by a Fourier infrared spectrometer (Spectrum 100, PerkinElmer Inc, Waltham, MA, USA) in a KBr pellet. The diameters of ten randomly selected P(St-BMA) microspheres and TTO microspheres were measured using a ruler. The surface morphology of the P(St-BMA) microspheres and the TTO microspheres were observed by optical microscopy (OLYMPUS C41, Olympus Corp, Tokyo, Japan).

### 3.4. Performance Testing

Thermal stability: the thermal properties of TTO, P(St-BMA) microspheres, and TTO microspheres were measured by thermogravimetric analysis (TGA 2, Mettler Toledo, Zurich Switzerland). Under nitrogen, the test temperature range was 40–500 °C with 10 °C/min heating intervals.

Oil absorbtion performance [35,36]: A certain weight amount (m_1_) of the P(St-BMA) microspheres was coated with a copper mesh and put into TTO at room temperature for 30 min. The microspheres were taken out, and the corresponding weights (m_2_) were measured before the oil droplet surface was allowed to drip freely for 5 min. The oil absorbency (*Q*) was calculated according to Equation (1) as follows:

(1) $$Q=\frac{m_{2}-m_{1}}{m_{1}}$$

Sustained-release performance: The TTO microspheres were tiled on a watch glass and TTO inside the microspheres was released in air atmosphere at 37 °C. The weight of TTO microspheres (m_i_) was measured at intervals of time. The cumulative release (*R*_i_) was calculated using Equation (2) as follows:

(2) $$R_{i}=\frac{m_{2}-m_{i}}{m_{2}-m_{1}}\times 100\%$$

Anti-bacterial activity experiment: *Escherichia coli* was cultured in Luria Bertani (LB) broth overnight at 37 °C. The bacterial solution was diluted with sterilized water. LB agar plates (90 mm) were prepared [37,38]. 100 μL of the bacterial solution was add to the plate, coated evenly with a spreader and hit a 6.0 mm hole by punch. 50 μL, 40 μL, 30 μL, and 20 μL of TTO and the corresponding oil dosage of the TTO microspheres were added to the hole, respectively, and cultured at 37 °C for 24 h. In addition, 50 μL of TTO, as well as the corresponding oil amount of the TTO microspheres, were allowed to naturally release TTO for 0 h, 12 h, 24 h, and 36 h, respectively. Afterwards, the amounts were added to the hole and cultured at 37 °C for 24 h. The diameter of inhibition zone was measured via the cross intersection method.

## 4. Conclusions

TTO can inhibit bacteria by volatilization, which is gradually being paid attention to. This application in food can effectively reduce the use of chemical antibacterial agents. Thus, the materials are very important to the load and sustained release of TTO. However, so far only a few studies on the adsorption and sustained release properties of TTO have been reported. Therefore, there is great potential for the development of materials that ensure the appropriate load and sustained release of TTO. Using butyl methacrylate and styrene as polymerization monomers, as well as diethylbenzene as a crosslinking agent, P(St-BMA) microspheres were prepared by suspension polymerization. The material has then been demonstrated to adsorb TTO to obtain TTO microspheres. The results showed that the stability of the TTO could be significantly improved after adsorption into P(St-BMA) microspheres. Upon control of the monomer ratio and the dosage of crosslinking agent, the oil absorption properties and sustained release properties could be adjusted. The sustained release system of the TTO microspheres follows the Korsmeyer-Pappas kinetic model, and the release of TTO from the TTO microspheres could mainly be explained by the Fickian diffusion model. The anti-bacterial effect of TTO microspheres and TTO was not significant at the same dosage of TTO. However, after 13 h of sustained release, the anti-bacterial effect of TTO microspheres was demonstrated to be significantly improved as compared to that of TTO.

## Figures and Tables

**Table 1:** FTIR spectra absorption peaks of TTO, P(St-BMA) microspheres, and TTO microspheres.

Functional Group	Wavelength (cm^−1^)		
	TTO	P(St-BMA) Microspheres	TTO Microspheres
O–H stretching vibration	3478.	-	3447
C–H (benzene ring) stretching vibration	-	2926, 1450	2926, 1452
C–H (C=C) stretching vibration	2965	-	2965
carbonyl stretching vibration	-	1729	1729
C–C (benzene ring) stretching vibration	-	1600	1600
C–O (ester) stretching vibration	1166	1174	1174
C–O (tertiary alcohol in terpenes) stretching vibration	1127	-	1127
C=C stretching vibration	902	-	910
C–H (benzene ring) plane bending vibration	-	763, 688	763, 689

**Table 2:** Fitting results for release curves at various monomer ratios.

Kinetic Model	Zero-Order	First-Order	Higuchi	Korsmeyer-Peppas	
Sample	*r* ^2^				*n*
m_St_:m_BMA_ = 2:8	0.7599	0.8863	0.9288	0.9733	0.3418
m_St_:m_BMA_ = 4:6	0.7365	0.8963	0.9221	0.9759	0.4446
m_St_:m_BMA_ = 6:4	0.7094	0.8487	0.9018	0.9718	0.4191
m_St_:m_BMA_ = 8:2	0.8049	0.8687	0.9567	0.9937	0.3275
m_St_:m_BMA_ = 10:0	0.8782	0.9383	0.9844	0.9886	0.4005
